# Supplementary material for: A conserved histidine modulates HSPB5 structure to trigger chaperone activity in response to stress-related acidosis
Source: eLife. 2015 May 11;4:e07304. doi: 10.7554/eLife.07304 (PMC4456606; doi:10.7554/eLife.07304)
Supplement: Figure 1—source data 1. — Backbone root mean square deviations (rmsd) between the solution structure and published structures and the correlation between experimentally measured residual dipolar coupling (RDC) values and those calculated for each published structure (PALES Correlation Coefficient) are given. Values in parentheses are calculated without including loop regions between the β-strands. Protomer–protomer and dimer–dimer rmsd between 2KLR and solution structure ensembles are the averages between all pairwise combinations of all structures in each ensemble. Rmsd values between the solution structure ensemble and the crystal structures are the average of all pairwise rmsd between the ten structures in the ensemble and the crystal structure. 2KLR: solid-state NMR structure; 2WJ7:crystal structure at pH 9.0; 3L1G:crystal structure at pH 4.6; 4M5S: crystal structure at pH 6.0. DOI: http://dx.doi.org/10.7554/eLife.07304.004 [file elife07304s001.docx]

| **Structure** | **Protomer-Protomer RMSD (Å)** | **Dimer-Dimer RMSD (Å)** | **PALES Correlation Coefficient** |
| --- | --- | --- | --- |
| 2KLR | 2.403 (1.637)* | 5.580 (3.90)* | 0.711 |
| 2WJ7 | 1.737 (1.04)* | 2.258 (2.042)* | 0.81 |
| 3L1G | 1.904 (1.088)* | 3.67 (3.275)* | 0.577 |
| 4M5S | 1.913 (1.164)* | 6.096 (4.77)* | 0.649 |

**Figure 1-Source data 1.** Root Mean Square Deviations (RMSDs) between the solution structure and published structures and the correlation between calculated and experimental RDC values (PALES Correlation Coefficient) are listed. 2KLR: Solid-state NMR structure; 2WJ7:crystal structure at pH 9.0; 3L1G:crystal structure at pH 4.6; 4M5S: crystal structure at pH 6.0.

*Values in parentheses indicate RMSDs calculated without including the loop regions between the β-strands. Protomer-protomer and dimer-dimer RMSDs between 2KLR and solution structure ensembles are the averages between all the pairwise combinations of all the structures in the ensemble. RMSDs between the solution structure ensemble and the crystal structures, 2WJ7, 3L1G, and 4M5S are the average of the all the pairwise RMSDs between the ten structures in the ensemble and the crystal structure.
