# Supplementary material for: A conserved histidine modulates HSPB5 structure to trigger chaperone activity in response to stress-related acidosis
Source: eLife. 2015 May 11;4:e07304. doi: 10.7554/eLife.07304 (PMC4456606; doi:10.7554/eLife.07304)
Supplement: Figure 1—source data 2. — ClustalOmega was used to align the sequences. Structural features of HSPB5-ACD are highlighted as follows: ACD is denoted by the blue line; His-104 is identified by the blue arrow; Loop 5/6 is shown in red box; dimer interface is shown in green box. All ACD histidine residues are shown in bold font. DOI: http://dx.doi.org/10.7554/eLife.07304.005 [file elife07304s002.pdf]

```
HSPB1  1    MTERRVPFSLLRGPSW-----DPFRDWYPHSRLF---DQA-------FGLPRLPEEWSQW  45
HSPB2  1    MSGRSVPHAHP---------ATAEYEFANPSRLG---EQR-------FGEGLLPEEILT-  40
HSPB3  1    MAKIILRHLI-----------------EIPVRYQ---EEF-------EARGLEDCRL---  30
HSPB4  1    -------MDVTIQHPWFKRTLGP---F-YPSRLF---DQF-------FGEGLFEYDLLPF  39
HSPB5  1    -------MDIAIHHPWIRRPFFP---FHSPSRLF---DQF-------FGEHLLESDLFPT  40
HSPB6  1    -----MEIPVPVQPSWLRRASAPLPGLSAPGRLF---DQR-------FGEGLLEAELAAL  45
HSPB7  1    MSHRT--------SSTF----RAERSFHSSSSSSSSTSSSASRALPAQDPPMEKALS--  46
HSPB8  1    MADGQMPFSCHYP-SRL--RRDPFRDSPLSSRLL---DDG-------FGMDPFPDDLTAS  47
HSPB9  1    ----------------MQ---RVGNTFSNESR------------------------VASR  17
HSPB11 1    MRKIDLCLSS----------------EGSEVILATSSDEKH------PPENIIDGNPETF  38

HSPB1  46   LG-------GSSWPGYVRPLPPAAIESPAVAAPAYSRALSRQLSSGVSEIRHTADRWRVS  98
HSPB2  41   -P-------TLYHGYYVRPRAAP---------------AGEGSRAGASELRLSEGKFQAF  77
HSPB3  31   -----------DHALYALPGPT--IVDLR---KTRAAQSPPVDSAAETPPREGKSHFQIL  74
HSPB4  40   LS-------STISPYY-RQ-------------SLF----RTVLDSGISEVRSDRDKFVIF  74
HSPB5  41   ST-------SLSPFYL-RP-------------PSFLR-APSWFDTGLSEMRLEKDRFSVN  78
HSPB6  46   CP-------TTLAPYYLRA-------------PSV--------ALPVAQVPTDPGHFSVL  77
HSPB7  47   -------MFSDDFGSFMRPHSEP--------------LAFPARPGGAGNIKTLGDAYEFA  85
HSPB8  48   WPDWALPRLSSAWPGTLRSGMVPR-------GPTA-TARFGVPAEGRTPPPFPGEPWKVC  99
HSPB9  18   CPS---------VGLAERNRVAT--------MPV---RLLRDSPAAQEDNDHARDGFQMK  57
HSPB11 39   WT---------------TTG-------------------------------MFPQEFIIC  52
                        ..   .  : : :                        :

HSPB1  99   LDVNHFAPDELTV------------------KTKDGVVEITGKHEE-------RQDEHG-  132
HSPB2  78   LDVSHFTPDEVTV------------------RTVDNLLEVSARHPQ-------RLDRHG-  111
HSPB3  75   LDVVQFLPEDIII------------------QTFEGWLLIKAQHGT-------RMDEHG-  108
HSPB4  75   LDVKHFSPEDLTV------------------KVQDDFVEIHGKHNE-------RQDDHG-  108
HSPB5  79   LDVKHFSPEELKV------------------KVLGDVIEVHGKHEE-------RQDEHG-  112
HSPB6  78   LDVKHFSPEEIAV------------------KVVGEHVEVHARHEE-------RPDEHG-  111
HSPB7  86   VDVRDFSPEDIIV------------------TTSNNHIEVRAEK----------LAADG-  116
HSPB8  100  VNVHSFKPEELMV------------------KTKDGYVEVSGKHEE-------KQQEGG-  133
HSPB9  58   LDAHGFAPEELVV------------------QVDGQWLMVTGQQQL-------DVRDPER  92
HSPB11 53   FHK-HVRIERLVIQSYFVQTLKIEKSTSKEPVDFEQWIEKDLVHTEGQLQNEEIVAHDGS  111
                                     :   ..       :  .* :

HSPB1  133  --YISRCFTRKYTLPPGVDPTQVSSSLSPEGTLTVEAPMPKLAT--QSN--EITIPVTFE  186
HSPB2  112  --FVSREFCRTYVLPADVDPWRVRAALSHDGILNLEAPRGGRHLDTEVN--EVYISLLPA  167
HSPB3  109  --FISRSFTRQYKLPDGVEIKDLSAVLCHDGILVVEVKDPVGTK----------------  150
HSPB4  109  --YISREFHRRYRLPSNVDQSALSCSLSADGMLTFCGPKIQTGLDATHA--ERAIPVSRE  164
HSPB5  113  --FISREFHRKYRIPADVDPLTITSSLSSDGVLTVNGPRKQV----SGP--ERTIPITRE  164
HSPB6  112  --FVAREFHRRYRLPPGVDPAAVTSALSPEGVLSIQAAPASAQAPPPAA--AK-------  160
HSPB7  117  --TVMNTFAHKCQLPEDVDPTSVTSALREDGSLTIRARRHPHTEHVQQ-TFRTEIKI---  170
HSPB8  134  --IVSKNFTKKIQLPAEVDPVTVFASLSPEGLLIIEAPQVPPYSTFGESSFNNELPQDSQ  191
HSPB9  93     VSYRMSQKVHRKMLPSNLSPTAMTCCLTPSGQLWVRGQCVALALPEAQTGP-------  143
HSPB11 112    ATYL------RFIIVSAFDHFASVHSVSAEGTVVSNLSS-------------------  144
```

```
HSPB1 187 SRAQLGGPEAAKSDETAAK---  205
HSPB2 168 -------PPDPEEEEEAAIVEP  182
HSPB3 151 ----------------------  150
HSPB4 165 EKPTSAPSS-------------  173
HSPB5 165 EKPAVTAAPKK-----------  175
HSPB6 161 ----------------------  160
HSPB7 171 ----------------------  170
HSPB8 192 E-------------VTCT----  196
HSPB9 144 -SPRLGSLGSKASNLTR-----  159
IFT25 145 ----------------------  144
```
