# Supplementary material for: A conserved histidine modulates HSPB5 structure to trigger chaperone activity in response to stress-related acidosis
Source: eLife. 2015 May 11;4:e07304. doi: 10.7554/eLife.07304 (PMC4456606; doi:10.7554/eLife.07304)
Supplement: Figure 2—source data 1. — DOI: http://dx.doi.org/10.7554/eLife.07304.009 [file elife07304s003.docx]

**Figure 2-Source data 1. Parameters from relaxation dispersion experiments performed on 0.7 mM HSPB5-ACD at 22 ºC and pH 7.5**

| **Residue** | ***k_ex_* [s^-1^]^a^** | **Model*^b^*** | ***p_a_^a^*** | **δω (^15^N ppm)^a^** | **χ^2^_red_** |
| --- | --- | --- | --- | --- | --- |
| 78 | 757.3±231.5 | 2 |  |  | 1.3 |
| 83 | 728.2±510.3 | 2 |  |  | 0.8 |
| 95 | 204.3±164.7 | 2 |  |  | 27.8 |
| 96 | 1826.1±892.5 | 2 |  |  | 16.1 |
| 102 | 831.6±481.0 | 3 | 97.0±8.3 | 2.0±0.6 | 2.7 |
| 106 | 628.7±501.8 | 3 | 97.9±11.5 | 1.5±0.8 | 19.8 |
| 108 | 177.1±242.8 | 3 | 94.1±11.6 | 0.7±0.5 | 47.9 |
| 109 | 252.3±921.9 | 3 | 88.3±15.0 | 8.3±1.5 | 2.9 |
| 114 | 1512.2±509.3 | 3 | 98.0±0.4 | 4.1±0.5 | 3.1 |
| 116 | 1978.6±917.1 | 3 | 97.8±3.2 | 10.0±0.7 | 2.6 |
| 117 | 928.7±290.4 | 3 | 95.1±5.9 | 1.9±0.4 | 3.8 |
| 118 | 797.5±394.7 | 3 | 94.1±14.6 | 1.6±0.5 | 2.7 |
| 119 | 1162.9±631.0 | 2 |  |  | 0.8 |
| 120 | 1498.3±1392.6 | 3 | 97.1±11.7 | 6.8±1.9 | 22.9 |
| 122 | 1119.3±547.6 | 2 |  |  | 7.6 |
| 136 | 768.0±699.5 | 3 | 97.6±6.1 | 3.7±0.8 | 10.7 |

*^a^*The exchange rate, *k*_ex_, *p*_a_, and δω are reported as fitted values ± standard deviation of the mean from 100 Monte Carlo simulations.

*^b^* The model numbers 2 and 3 refer to two different exchange regimes: 1) fast exchange (model-2, *k*_ex_ >> δω) and 2) slow exchange (model-3, *k*_ex_ << δω).
