# Supplementary material for: A conserved histidine modulates HSPB5 structure to trigger chaperone activity in response to stress-related acidosis
Source: eLife. 2015 May 11;4:e07304. doi: 10.7554/eLife.07304 (PMC4456606; doi:10.7554/eLife.07304)
Supplement: Figure 4—source data 1. — n.d.: not determined because resonances broaden below pH 7.5 and become undetectable. DOI: http://dx.doi.org/10.7554/eLife.07304.013 [file elife07304s004.docx]

| **Residue** | **p*K*_r_** | **Tautomer** |
| --- | --- | --- |
| 83 | 6.6 | Nε2H |
| 101 | < 6 | Nε2H |
| 104 | n.d*^a^*. | Nδ1H |
| 111 | < 5 | Nε2H |
| 119 | 7.7 | Nε2H |

**Figure 4-Source data 1. The p*K*_r_ values and tautomeric states of Histidine side-chain imidazole rings at 22 ºC are listed.**

*^a^*n.d.: not determined since the resonances broaden out below pH 7.5.
